# RETRACTION: HSP90 Inhibition Suppresses Inflammatory Response and Reduces Carotid Atherosclerotic Plaque Formation in ApoE Mice

**DOI:** 10.1155/cdr/9796386

**Published:** 2026-06-03

**Authors:** Cardiovascular Therapeutics

RETRACTION: H. Mu, L. Wang, L. Zhao, “HSP90 Inhibition Suppresses Inflammatory Response and Reduces Carotid Atherosclerotic Plaque Formation in ApoE Mice,” Cardiovascular Therapeutics (2016): e12243, https://doi.org/10.1111/1755-5922.12243

The above article, published online on 23 December 2016 in Wiley Online Library (wileyonlinelibrary.com), has been retracted by agreement between the Journal Editor‐in‐Chief, Baohui Xu; and John Wiley & Sons Ltd. The retraction has been agreed due to multiple concerns identified with the figures, initially identified on PubPeer [1]. Subsequent investigation by the publisher identified further instances of image duplication and similarities to panels and Western Blots in figures of previously published articles [2–5], specifically concerning Figures 1a, 2b, 3a, 4a, 4b, 4c, 5a and 5b.

After assessment of the concerns, the Chief Editor has determined that the results and conclusions are no longer reliable, and therefore the article is retracted.

The authors did not provide a response to the concerns or the retraction.
